# Rydberg interaction induced enhanced excitation in thermal atomic vapor

**DOI:** 10.1038/s41598-018-23559-0

**Published:** 2018-03-27

**Authors:** Dushmanta Kara, Arup Bhowmick, Ashok K. Mohapatra

**Affiliations:** School of Physical Sciences, National Institute of Science Education and Research Bhubaneswar, HBNI, Jatni, 752050 India

## Abstract

We present the experimental demonstration of interaction induced enhancement in Rydberg excitation or Rydberg anti-blockade in thermal atomic vapor. We have used optical heterodyne detection technique to measure Rydberg population due to two-photon excitation to the Rydberg state. The anti-blockade peak which doesn’t satisfy the two-photon resonant condition is observed along with the usual two-photon resonant peak which can’t be explained using the model with non-interacting three-level atomic system. A model involving two interacting atoms is formulated for thermal atomic vapor using the dressed states of three-level atomic system to explain the experimental observations. A non-linear dependence of vapor density is observed for the anti-blockade peak which also increases with increase in principal quantum number of the Rydberg state. A good agreement is found between the experimental observations and the proposed interacting model. Our result implies possible applications towards quantum logic gates using Rydberg anti-blockade in thermal atomic vapor.

## Introduction

Long range many body interaction in Rydberg atoms give rise to many interesting phenomena. The suppression in Rydberg population or the excitation blockade is the most striking one giving rise to a variety of applications^1^. A highly dense atomic ensemble behaves like a single super atom producing a strongly correlated many body system^2^ and also leading to a single photon source^3^. The phenomenon has been experimentally observed in an atomic ensemble in a magneto optical trap^4–7^, in a magnetic trap^8,9^ and also in a single atom trap^10,11^. Many theoretical models focus on the study of strongly correlated many body system in ultra cold atom and Bose Einstein condensate^12–15^. Rydberg blockade interaction can induce optical non-linearity which is non-local^16^ and also strong enough to observe for single photon^17,18^. Rydberg blockade interaction may also lead to applications such as quantum gates using atoms^19–22^. An opposite effect of Rydberg blockade with enhancement in Rydberg excitation facilitated by interaction called as the Rydberg anti-blockade has been proposed in ultra cold atomic gas using a two photon excitation to Rydberg state^23^. An experiment performed in ultra cold ensemble of atoms verifies the effect based on the theoretical model^24^. It has been proposed that resonant dipole dipole interaction has non-additive character due to anti-blockade in an ensemble having more than two atoms in the blockade sphere^25^. In addition to this, the existence of anti-blockade between two Rydberg atoms, interacting with a zero area phase jump pulse is also reported^26^. Some recent results have also been reported to study Rydberg anti blockade in atomic^27–29^ and molecular resonances^30^. The implementation of quantum logic gate using Rydberg anti-blockade has also recently been proposed^31–33^.

Recent experiments with thermal vapor have drawn the attention to study Rydberg interaction induced manybody effects^34–38^. Electromagnetically induced transparency involving Rydberg state in thermal vapor cell as well as in micron size vapor cell has been studied^39,40^. In addition, four wave mixing for a Rydberg state^41^ and kerr non-linearity in Rydberg EIT has also been reported in thermal Rubidium vapor^42^. A recent study of Rydberg blockade in thermal atomic vapor has also been performed^43^. Anomalous excitation facilitated by Rydberg interaction has also been proposed recently in thermal atomic vapor^44^. In this article, we present a strong evidence of enhancement in Rydberg excitation due to interaction in thermal atomic vapor. An interacting twoatom model is formulated using the dressed state picture of a three level system in cold atomic ensemble. The model is further extended to thermal atomic vapor by Doppler averaging over the ensemble. An experiment has been performed in thermal rubidium vapor using optical heterodyne detection technique^45^ to observe the anti-blockade effect. A good match is found between the model and the experimental observation as an evidence of the existence of Rydberg anti-blockade in thermal atomic vapor. This is the first ever direct spectroscopic observation of Rydberg anti-blockade in thermal atomic vapor.

## Results

### Theoretical model

**Figure 1:**
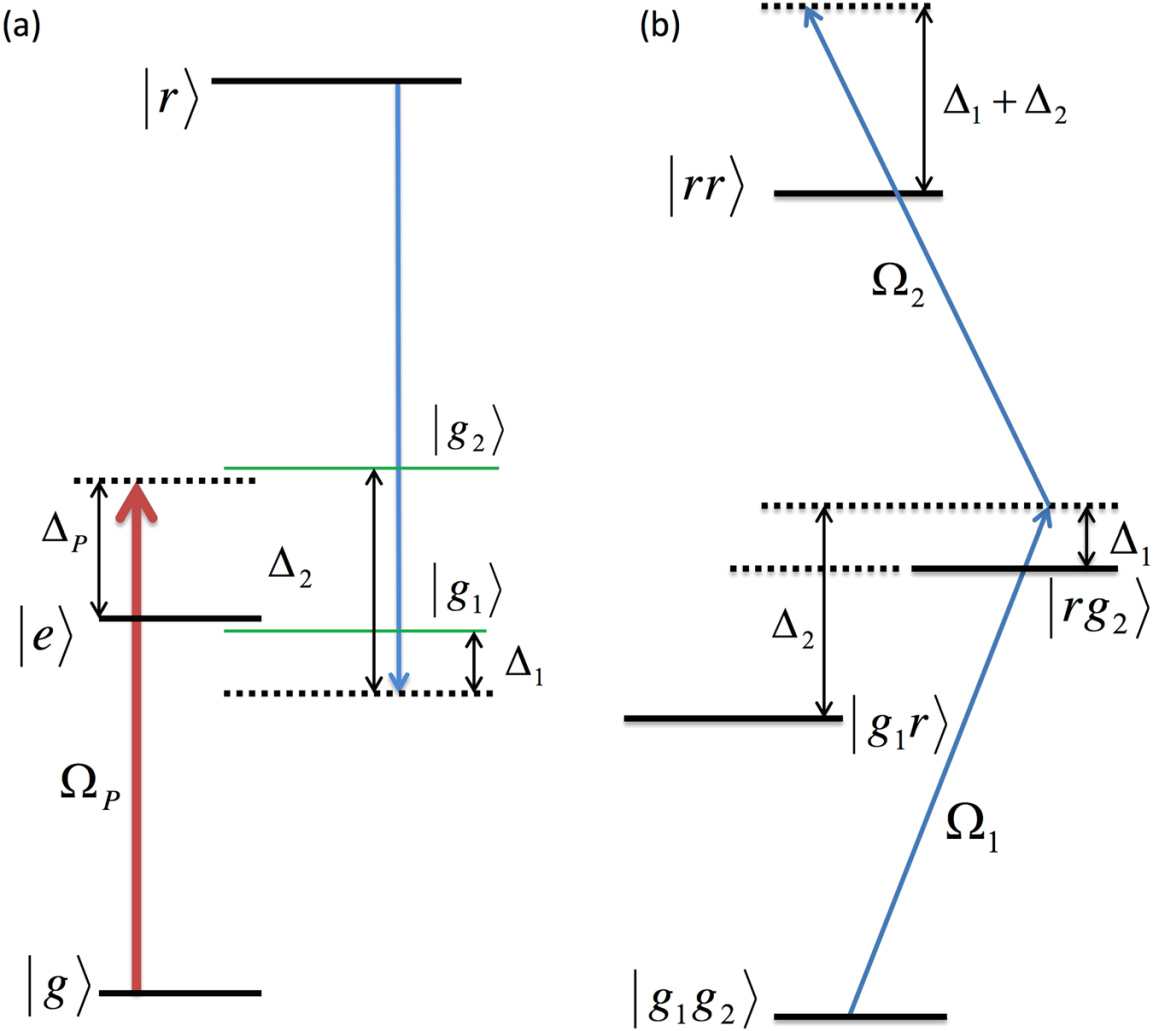
The relevant energy level diagrams. (**a**) A probe laser driving the |*g*〉→|*e*〉 transition of a single atom leads to the dressed states |*g*_1_〉 and |*g*_2_〉. The coupling laser drives the dressed states to the Rydberg state |*r*〉 with detuning ${{\rm{\Delta }}}_{1}\simeq {{\rm{\Delta }}}_{C}-{{\rm{\Omega }}}_{P}^{2}\mathrm{/4}{{\rm{\Delta }}}_{P}$ and ${{\rm{\Delta }}}_{2}\simeq {{\rm{\Delta }}}_{P}+{{\rm{\Delta }}}_{C}+{{\rm{\Omega }}}_{P}^{2}\mathrm{/4}{{\rm{\Delta }}}_{P}$ respectively. (**b**) The twoatom model with states |*g*_1_*g*_2_〉 representing an atom in each dressed states, |*g*_1_*r*〉, |*rg*_2_〉 representing one atom in the respective dressed state and other atom in the Rydberg state and |*rr*〉 representing both the atoms in the Rydberg state.

Let us consider a three level atomic system with states |*g*〉, |*e*〉 and |*r*〉 as shown in Fig. 1(a). The probe (coupling) laser drives the transition |*g*〉→|*e*〉 (|*e*〉→|*r*〉) with Rabi frequency Ω_*p*_ (Ω_*C*_) and detuning Δ_*P*_ (Δ_*C*_). Both of these transitions are dipoleallowed whereas the transition |*g*〉→|*r*〉 is dipoleforbidden. The population decay rates of the transitions |*e*〉→|*g*〉 and |*r*〉→|*e*〉 are Γ_*eg*_ and Γ_*re*_ respectively. We have also included a decay rate Γ_*rg*_ to account for the indirect decay of the state |*r*〉→|*g*〉 due to finite transit time of the thermal atoms. In the regime Ω_*C*_ ≪ Ω_*P*_, interaction with the coupling laser can be treated as a small perturbation to the atomic states dressed by the strong probe laser. For large probe detuning Δ_*P*_ ≫ Ω_*P*_ and Γ_*eg*_, the dressed states are given by $|{g}_{1}\rangle \approx |e\rangle +\frac{{{\rm{\Omega }}}_{P}}{2{{\rm{\Delta }}}_{P}}|g\rangle$ and $|{g}_{2}\rangle \approx |g\rangle -\frac{{{\rm{\Omega }}}_{P}}{2{{\rm{\Delta }}}_{P}}|e\rangle$ with difference in their energy eigenvalues ${\rm{\Delta }}\simeq {{\rm{\Delta }}}_{P}+{{\rm{\Omega }}}_{P}^{2}\mathrm{/2}{{\rm{\Delta }}}_{P}$. The steady state population of the dressed states can be determined by diagonalizing the steady state density matrix for the two-level atomic transition |*g*〉→|*e*〉 driven by the strong probe laser. The population of the states |*g*_1_〉 and |*g*_2_〉 are found to be approximately ${{\rm{\Omega }}}_{P}^{4}\mathrm{/16}{{\rm{\Delta }}}_{P}^{4}$ and 1 respectively. When the coupling laser is scanned over these dressed states, each of them will behave like a ground state exciting to the Rydberg state by the coupling laser. It is to be noted that the optical pumping rate to achieve the steady state population of the dressed states is Γ_*eg*_. If Ω_*C*_ ≪ Γ_*eg*_ then the coupling laser driving to the Rydberg state can not build the coherence between the dressed states. Hence, both the states can be treated independently and the total Rydberg population can be determined by adding the individual Rydberg populations driven from each of the dressed states. The coupling Rabi frequencies are scaled for each dressed state as Ω_1_ ≈ Ω_*C*_ for the transition |*g*_1_〉→|*r*〉 and ${{\rm{\Omega }}}_{2}\approx {{\rm{\Omega }}}_{P}{{\rm{\Omega }}}_{C}\mathrm{/2}{{\rm{\Delta }}}_{P}$ for the transition |*g*_2_〉→|*r*〉. Similarly, the population of the Rydberg state will decay to the states |*g*_1_〉 and |*g*_2_〉 with decay rates as ${{\rm{\Gamma }}}_{1}\simeq {{\rm{\Gamma }}}_{re}+\frac{{{\rm{\Omega }}}_{P}}{2{{\rm{\Delta }}}_{P}}{{\rm{\Gamma }}}_{rg}$ and ${{\rm{\Gamma }}}_{2}\simeq {{\rm{\Gamma }}}_{rg}+\frac{{{\rm{\Omega }}}_{P}}{2{{\rm{\Delta }}}_{P}}{{\rm{\Gamma }}}_{re}$ respectively.

**Figure 2:**
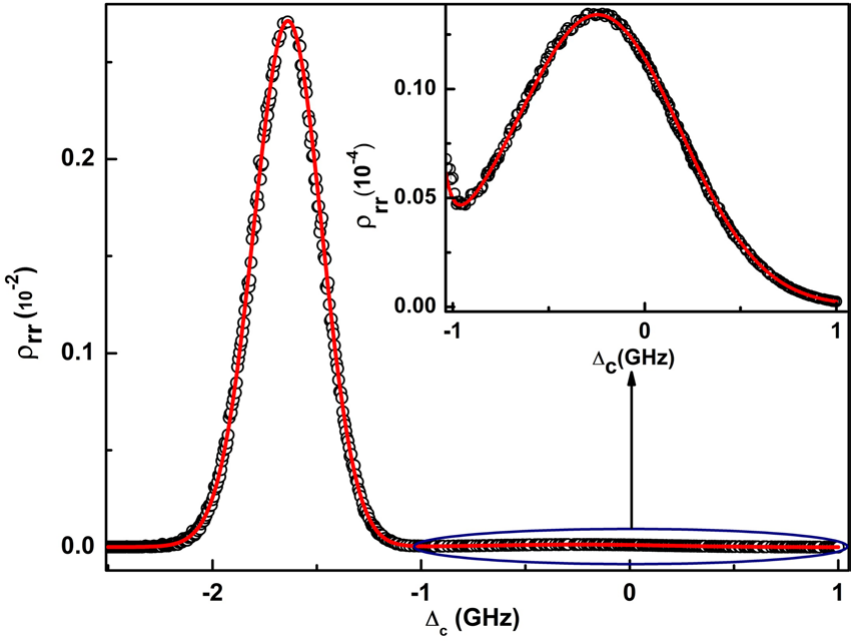
Rydberg population as a function of coupling laser detuning. Rydberg population calculated using exact three level single atomic system and two-atoms non-interacting model are represented by the solid line and the symbol (°) respectively. Inset shows the magnified view of the peak near Δ_*C*_ = 0. Laser parameters used in the calculation are Ω_*P*_ = 400 MHz, Ω_*C*_ = 5 MHz and Δ_*P*_ = 1.25 GHz.

Considering two atoms driven to the Rydberg state simultaneously, there are four possible dressed states as |*g*_1_*g*_1_〉, |*g*_2_*g*_2_〉, |*g*_1_*g*_2_〉 and |*g*_2_*g*_1_〉. The states |*g*_1_*g*_2_〉 and |*g*_2_*g*_1_〉 are degenerate and have equal steady state population of ${{\rm{\Omega }}}_{P}^{4}\mathrm{/16}{{\rm{\Delta }}}_{P}^{4}$. The population of the state |*g*_2_*g*_2_〉 is approximately 1 and that of |*g*_1_*g*_1_〉 is negligibly small for ${{\rm{\Omega }}}_{P}\ll {{\rm{\Delta }}}_{P}$. The difference in the energy eigenvalues of |*g*_2_*g*_2_〉 and |*g*_1_*g*_2_〉 is Δ which can be made larger than the typical Doppler linewidth of the transition in thermal vapor. If a narrow band laser is made resonant to the transition |*g*_1_*g*_2_〉→|*rg*_2_〉, then the laser will be out of resonance to drive the atoms in the state |*g*_2_ *g*_2_〉 to the Rydberg state. In the regime Ω_*C*_ ≪ Γ_*eg*_, the coupling laser can’t introduce coherence between the states |*g*_1_ *g*_2_〉 and |*g*_2_ *g*_1_〉. Hence, either of the states can be considered in the two atom model to determine the Rydberg population with proper normalization accounting for both the states. Thus in the simplified model, only one of the dressed states of the two atomic system can be considered to model the anti-blockade peak. As shown in Fig. 1(b), the relevant energy level diagram to model the anti-blockade peak are |*g*_1_*g*_2_〉, |*g*_2_*r*〉, |*rg*_2_〉 and |*rr*〉. Steady state solutions of the master equation using the above simplified model and averaging over the thermal ensemble (see method section) is depicted in Fig. 2. In the regime Ω_*C*_ ≪ Γ_*eg*_, the non-interacting two-atom dressed state model matches excellent with the exact three-level single atom calculation which tends to deviate with increase in Ω_*C*_ above Γ_*eg*_. It can be shown that the |*g*_2_〉→|*r*〉 transition is equivalent to the effective two-level transition (|*g*〉→|*r*〉) by adiabatically eliminating the intermediate state |*e*〉 of a three-level system^46^. The Rydberg population due to the |*g*_1_〉→|*r*〉 transition which is neglected in the model with effective two-level system can be shown to be enhanced due to Rydberg-Rydberg interaction. Hence, the exact model for three-level system for two interacting atoms is necessary to study the anti-blockade peak. However, for the given laser parameters discussed above, the dressed state model is simplified due to reduced Hilbert space relevant for modelling the anti-blockade peak.

**Figure 3:**
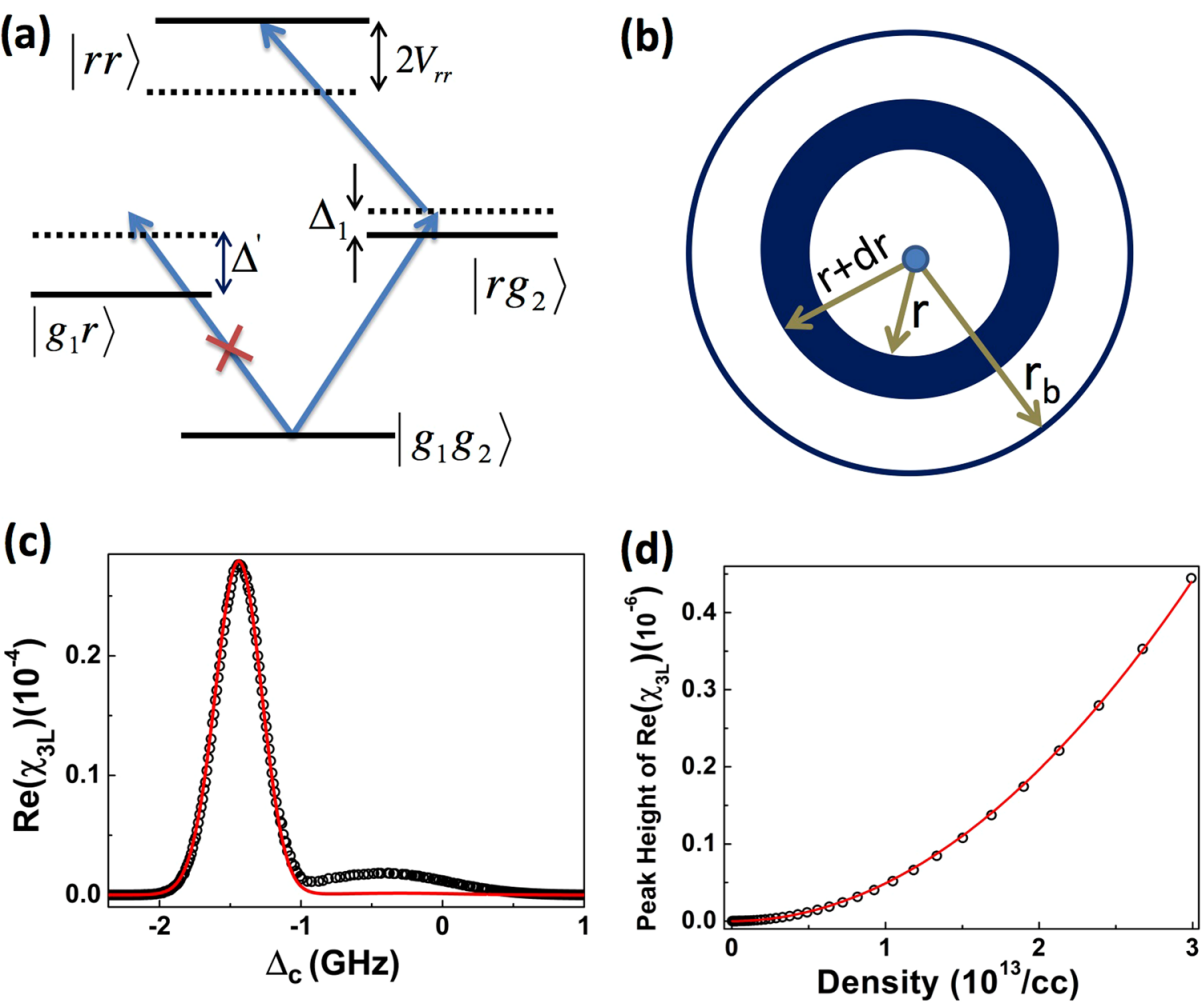
(**a**) Energy level diagram to model Rydberg anti-blockade. Δ_1_ and Δ_2_ (=Δ_1_ + Δ′) are the coupling laser detuning of atom 1 and 2 respectively. (**b**) Schematic of the interaction sphere of radius *r*_*b*_. The atom in the dressed state |*g*_2_〉 is placed at the centre of the sphere. The atoms in the spherical shell of radius *r* and thickness *dr* can compensate for the two-photon resonance to the |*rr*〉 state due to van der Waals type Rydberg-Rydberg interaction (*V*_*rr*_ = *C*_6_/*r*^6^) with the atom at the center. (**c**) Dispersion spectrum of the probe laser calculated using non-interacting model (solid line) and two-atoms interacting model (black open circle). (**d**) Calculated dispersion peak height of the anti-blockade peak using two-atoms interacting model (black open circle) showing the quadratic dependance (solid line) of density.

Rydberg-Rydberg interaction can easily be introduced in the model by including the shift in energy of the |*rr*〉 state. Rydberg anti-blockade in thermal vapor is illustrated in Fig. 3. Consider a case where the narrow band laser is resonant to the |*g*_1_*g*_2_〉→|*rg*_2_〉 transition as depicted in Fig. 3(a). Then the atom in the dressed state |*g*_1_〉 is excited to the Rydberg state. The same laser will be out of resonance to the |*g*_1_*g*_2_〉→|*g*_1_*r*〉 transition. If the Rydberg-Rydberg interaction is absent then the state |*rr*〉 will also not satisfy the resonant condition. Therefore, the second atom in the dressed state |*g*_2_〉 can’t be excited to the Rydberg state. Suppose the interaction shift of the |*rr*〉 state is equal to Δ′ (difference in the resonant frequencies corresponding to the |*g*_1_*g*_2_〉→|*rg*_2_〉 and |*g*_1_*g*_2_〉→|*g*_1_*r*〉 transitions) then the |*rr*〉 state will be resonant to the laser as shown in Fig. 3(a). Now the second atom present in the state |*g*_2_〉 will also be excited to the Rydberg state unlike a non-interacting system. So the presence of the Rydberg interaction facilitate the excitation of the second atom enhancing the total Rydberg population compared to the non-interacting case and this phenomenon is known as Rydberg anti-blockade. Rydberg anti-blockade peak appears when the coupling laser is resonant to the |*g*_1_〉→|*r*〉 transition i.e. near Δ_*C*_ = 0, whereas the usual two-photon resonant peak appears with the coupling laser resonating to the |*g*_2_〉→|*r*〉 transition. Referring to Fig. 3(b), consider an interaction sphere with radius *r*_*b*_ where *r*_*b*_ is defined as the blockade radius and is given by ${r}_{b}=\sqrt[6]{\frac{{C}_{6}}{h{{\rm{\Omega }}}_{2}}}$, with *C*_6_ being the coefficient of van der Waals interaction. Consider the atom in the dressed state |*g*_1_〉 resonating to the coupling laser to be at the center of the sphere. Assume that the second atom in the dressed state |*g*_2_〉 is present in a concentric spherical shell with radius |*r*〉 and thickness *dr*. Referring to Fig. 3(a), if Δ_1_ + Δ′ = 2*C*_6_/*r*^6^, then both the atoms will be excited to the |*rr*〉 state which will lead to the enhanced Rydberg excitation or anti-blockade. In a thermal atomic ensemble, effect of the atomic velocity distribution should be included in the detuning Δ_1_ and also in Δ′. Hence, in thermal atomic vapor, not all but only a specific velocity class of atoms in the given spherical shell of radius |*r*〉 can satisfy the resonance condition to the |*rr*〉 state. Then, the Rydberg population due to anti-blockade can be evaluated by averaging over the velocity distribution of the thermal ensemble and integrating over the interaction sphere (see method section). It is to be noted that the motion of the atom due to Rydberg-Rydberg interaction (e.g. for n = 50 state) is of the order of 100 nm while transiting through the laser beam of size 100 *μ*m which can be neglected. In a regime Ω_*P*_ ≫ Ω_*C*_, the dispersion of the probe beam is shown to be proportional to the Rydberg population^45^. So, the probe dispersion spectrum by changing the coupling laser detuning was calculated from the theoretical model which is shown in Fig. 3(c). However, the model will explain only the anti-blockade peak as the laser was resonant to the transition |*g*_1_*g*_2_〉→|*rg*_2_〉. The usual two photon resonant peak can be determined by considering excitation from the ground state |*g*_2_*g*_2_〉. For a comparison, the exact 3-level single atom calculation is also depicted in the same figure. Anti-blockade peak is observed to be enhanced significantly due to interaction compared to the non-interacting case. Referring to eq. (1) in method section, the dispersion of the probe beam due to two atoms interaction depends strongly on the principal quantum number of the Rydberg state and also depends quardratically on the density of the atomic vapor. The dispersion peak height of the anti-blockade peak calculated from the interacting model showing a quadratic dependence on the density of the atomic vapor is depicted in Fig. 3(d).

### Experimental results and discussions

**Figure 4:**
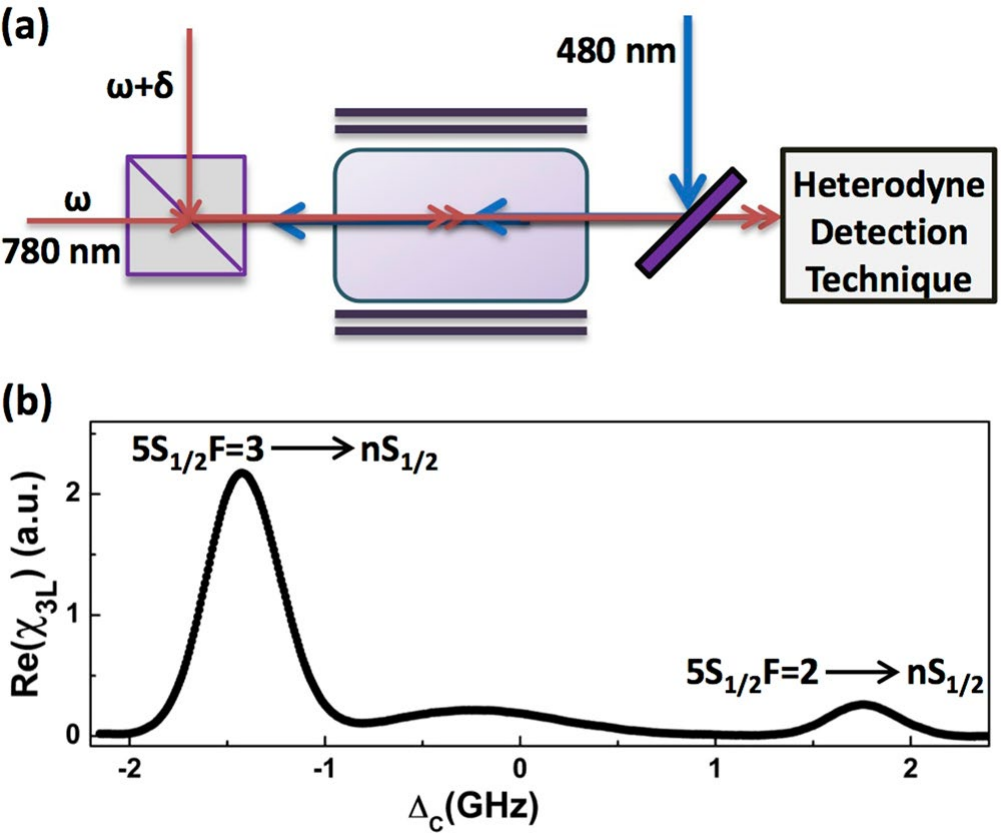
(**a**) Schematic of the experimental set up. (**b**) A typical dispersion spectrum of the probe laser observed using the optical heterodyne detection technique showing the resonant peaks corresponding to 5S_1/2_F = 3→*n*S_1/2_ and 5S_1/2_F = 2→*n*S_1/2_ of ^85^Rb.

Schematic of the experimental set up is depicted in Fig. 4(a). Optical heterodyne detection technique (OHDT)^42,45^ was used to measure the dispersion of the probe beam propagating through a magnetically shielded rubidium vapor cell. The details of the OHDT and the theoretical model for relating the dispersion with Rydberg population can be found in ref.^45^. Optical heterodyne detection technique requires a probe laser beam along with a reference laser beam which were derived from an external cavity diode laser operating at 780 nm. A frequency offset of 800 MHz between the probe and the reference beams was introduced using acousto-optic modulators. The coupling laser beam operating in the range of 478 nm to 482 nm counter-propagates the probe beam through the vapor cell. The overlapping between the probe and coupling beam was ensured by optimizing the Rydberg EIT signal. The beam waist of the probe (coupling) laser was 95 *μ*m (80 *μ*m) and the respective Rayleigh range was 36.33 mm (41.86 mm). The probe laser power was kept fixed at 4 mW throughout the experiment. For the coupling laser the power was varied following the *n*^3/2^ law, so as to keep the coupling rabi frequency constant for all the n states. The Rabi frequencies of the probe and coupling beams were determined from their intensity using the method discussed in ref^42^. The inhomogeneity of the laser intensity profile is neglected in the model and the averaged Rabi frequencies were used to compare with the experimental observation. The density of the vapor was varied by heating the cell and the temperature was controlled using a PID controller. The non-linear phase shift of the probe laser due to two-photon excitation to the Rydberg state in the presence of the coupling laser can be measured by comparing the phase of the reference beam using OHDT^45^. A typical dispersion spectrum observed in the experiment is shown in Fig. 4(b).

**Figure 5:**
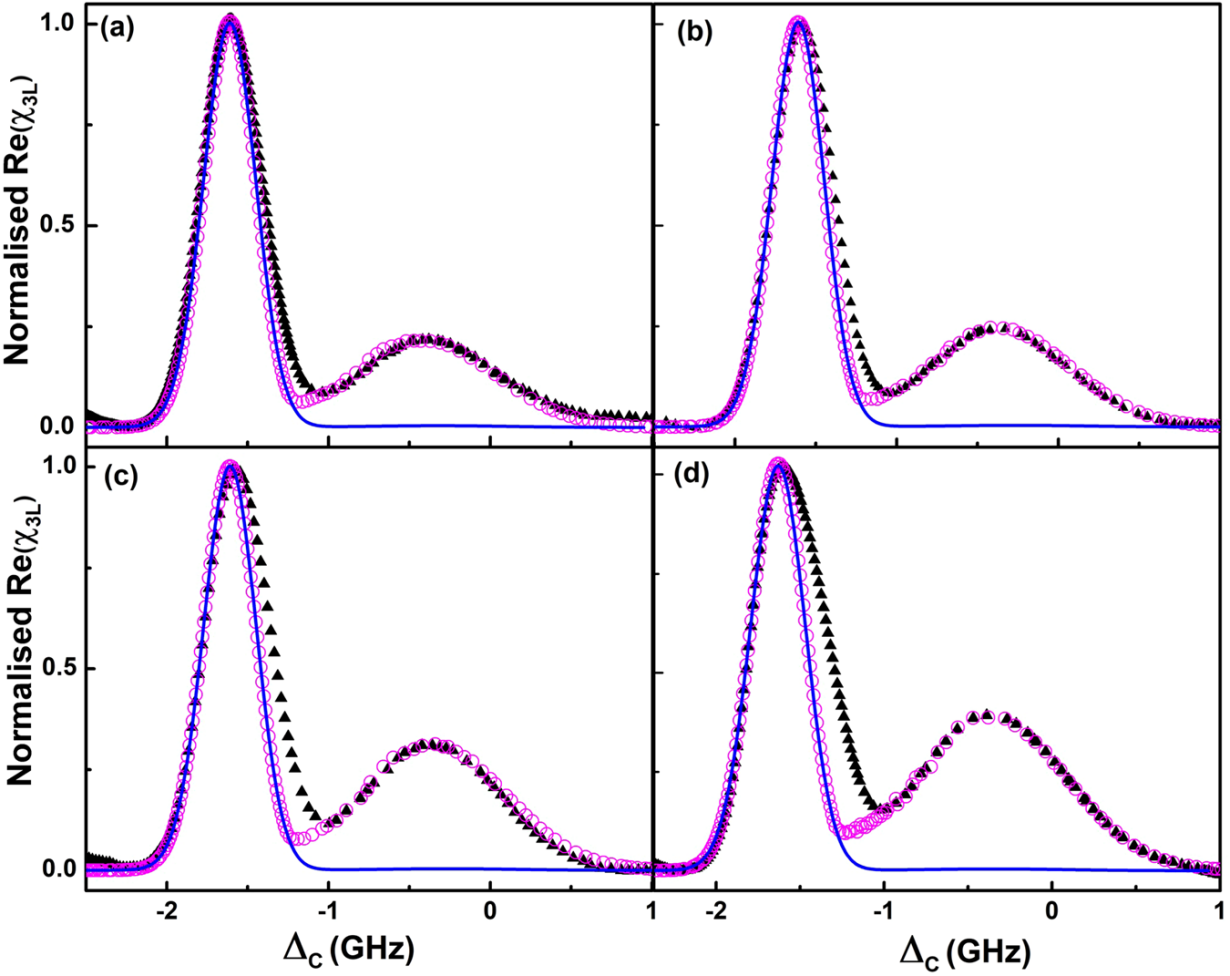
Dispersion spectrum measured from the experiment (black triangle) and calculated from the interacting two-atom model (open circle) for the Rydberg state with principal quantum numbers (**a**) *n* = 35 (**b**) *n* = 40 (**c**) *n* = 45 and (**d**) *n* = 53. For comparison, dispersion calculated from the non-interacting model is depicted as solid lines for all the *n* states.

The experiment was performed for the Rydberg states 35S_1/2_, 40S_1/2_, 45S_1/2_ and 53S_1/2_. The dispersion of the probe beam was measured using OHDT by varying the density of the rubidium vapor with laser parameters Ω_*P*_ = 400 MHz, Ω_*C*_ = 4 MHz and Δ_*P*_ = 1.25 GHz. All the laser parameters including the gain in the set up were kept fixed throughout the experiment for all the Rydberg states. As predicted in the theoretical model, two different peaks were observed for the dispersion spectrum of the probe beam when the coupling laser is scanned over few GHz. One of them is the usual two photon resonant peak and the other one is the anti-blockade peak. Since the Rydberg-Rydberg interaction is repulsive, the anti-blockade peak is expected to be observed on the blue detuned side of the dispersion spectrum. For lower principal quantum number states the Rydberg interactions is weak and is significant only at very high atomic density. However, with increase in the principal quantum number of the Rydberg state, the interaction is significant and hence the anti-blockade peak is observed at lower densities as well. The width of the two photon resonant peak is nearly Δ*kv*_*p*_, while for the anti-blockade peak, it is about *k*_*C*_*v*_*p*_. The width of the anti-blockade peak is observed to be larger which seems to be in good agreement with the theoretical model as shown in Fig. 5. For repulsive interaction, as shown in Fig. 3(a), when the laser is red detuned, the contribution of the off resonant atom to the Rydberg population is less as compared to a blue detuned case. Referring to the two photon resonant peak, the anti-blockade effect is significantly larger on the blue detuned side compared to the red detuned side while coupling to *n*S_1/2_ state. The two photon resonant peak contains both blockade and anti-blockade effect making it difficult to model. However, to have a qualitative understanding, this peak is compared to a non-interacting model. As shown in Fig. 5, with increase in principal quantum number, the experimental data deviates more from the noninteracting model. The deviation on the blue detuned side of the spectrum is an indication of the dominating anti-blockade effect.

**Figure 6:**
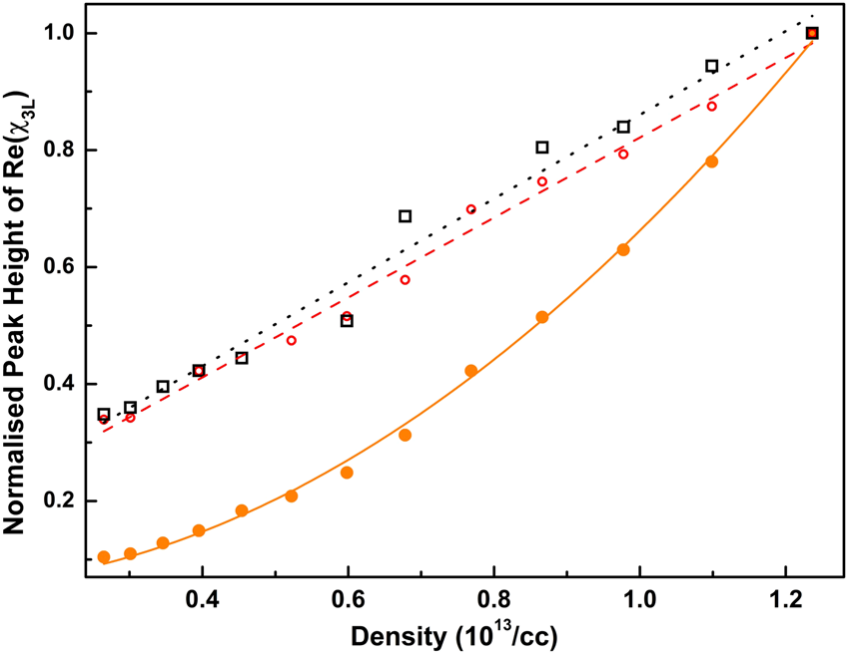
Anti-blockade peak height (solid circle) and the usual two-photon resonant peak height for ^85^Rb 5S_1/2_F = 3→*n*S_1/2_ (open circle) and ^85^Rb 5S_1/2_F = 2→*n*S_1/2_ transition(◊) for *n* = 35 Rydberg state as a function of the density of the atomic vapor. The peak heights are normalized to the dispersion peak height corresponding to the highest density. Dotted lines are the straight line fitting of usual two-photon resonant peaks showing the linear dependence of density whereas the solid line is fitting of the anti-blockade peak height showing quadratic dependence of density.

For a fixed atomic density, the height of the anti-blockade peak increases with the principal quantum number of the Rydberg state. The *C*_6_ scaling with the principal quantum number could not be determined from the anti-blockade peak observed in the experiment. When the number of atoms in the |*g*_2_〉 state in the interaction sphere is more than one, the blockade effect will contribute along with the cascaded processes involving more number of atoms. For a Rydberg state with n = 35, the interaction is small and the number of atoms in the |*g*_2_〉 state in the interaction sphere, *N*_*b*_ ≃ 1 at a density of 3.0 × 10^13^/*cc*. Thus, the experimental data is expected to match well with the model. We measured the dispersion peak height for the anti-blockade peak and the two photon resonant peaks corresponding to the transitions ^85^Rb 5S_1/2_ *F* = 2→35S_1/2_ and ^85^Rb 5S_1/2_ *F* = 3→35S_1/2_ by varying the density of the vapor cell which is shown in Fig. 6. For 35S_1/2_, the anti-blockade peak height increases quadratically with increase in density as predicted in the model. For the other two peaks, the variation is observed to be linear. The peak corresponding to the transition ^85^Rb 5S_1/2_ *F* = 2→35S_1/2_ is expected to be non-interacting as the applied laser is highly detuned from the atomic resonance resulting in linear dependence of density. For the two photon resonant peak corresponding to ^85^Rb 5S_1/2_ *F* = 3→35S_1/2_, both the blockade and anti-blockade effects are present which may be compensating each other such that the variation with density is roughly linear. The dotted and dashed lines are the linear fittings and the solid line is the quadratic fit of the peak height data as shown in Fig. 6.

## Conclusion

We have observed the interaction induced enhancement in Rydberg excitation in thermal rubidium vapor. A two atom interacting model is formulated using the dressed state picture of the three level atomic system to explain the anti-blockade peak. The population of the Rydberg state is observed to be enhancing quardratically with the density of the vapor for Rydberg state with n = 35, as predicted in the theoretical model. The experiment performed here is limited by the uncertainty in the density measurement. The density dependence of the anti-blockade peak can be studied with a better measurement of density and larger number of data. The deviation from the quadratic behavior can be measured to study the effect of blockade and the cascaded processes on the anti-blockade peak having more than one atom in the |*g*_2_〉 state in the interaction sphere.

## Methods

### Non-interacting dressed state model

The Hamiltonian for driving two atoms together is given by $$H={H}^{\mathrm{(1)}}\otimes {\bf{I}}+{\bf{I}}\otimes {H}^{\mathrm{(2)}}$$, where $${H}^{\mathrm{(1)}}=\frac{-\hslash }{2}({\rm{\Delta }}|{g}_{1}\rangle \langle {g}_{1}|{+{\rm{\Delta }}}_{1}|r\rangle \langle r|+{{\rm{\Omega }}}_{1}|{g}_{1}\rangle \langle r|+H.C.)$$ and $${H}^{\mathrm{(2)}}=\frac{-\hslash }{2}({{\rm{\Delta }}}_{2}|r\rangle \langle r|+$$$${{\rm{\Omega }}}_{2}|{g}_{2}\rangle \langle r|+H.C.)$$ are the Hamiltonian for the individual atoms. The Lindblad operator for the two-atom model can be written as $${ {\mathcal L} }_{D}={ {\mathcal L} }_{D}^{\mathrm{(1)}}\otimes {\rho }^{\mathrm{(2)}}+{\rho }^{\mathrm{(1)}}\otimes { {\mathcal L} }_{D}^{\mathrm{(2)}}$$, where $${ {\mathcal L} }_{D}^{(j)}={{\rm{\Gamma }}}_{j}(C{\rho }^{(j)}{C}^{\dagger }-\frac{1}{2}({C}^{\dagger }C{\rho }^{(j)}+{\rho }^{(j)}{C}^{\dagger }C))$$$$-\frac{{{\rm{\Gamma }}}_{eg}}{2}({C}^{\dagger }C{\rho }^{(j)}C{C}^{\dagger }+H.C.)$$, where *j* = 1,2 for atom 1 and atom 2 respectively and *C* = |*f*〉〈*i*|, where |*i*〉 stands for the initial state from which the population decays to the final state |*f*〉^47^. *ρ*^(1)^ and *ρ*^(2)^ represent the density matrices of atom 1 and 2 respectively. Rydberg population in the system can be determined from the steady state solution of the master equation $$\dot{\rho }=\frac{1}{i\hslash }[H,\rho ]+{ {\mathcal L} }_{D}$$. Rydberg population can be determined as $${\rho }_{rr}=(\frac{{\rho }_{22}+{\rho }_{33}}{2}+{\rho }_{44})\frac{{{\rm{\Omega }}}_{P}^{4}}{8{{\rm{\Delta }}}_{P}^{4}}$$ where the factor $$\frac{{{\rm{\Omega }}}_{P}^{4}}{8{{\rm{\Delta }}}_{P}^{4}}$$ is taken as the population of the state |*g*_1_*g*_2_〉 including the normalization factor to account for the population of the state |*g*_2_ *g*_1_〉. To model the system for thermal atomic vapor, both the atoms are considered to be moving with independent velocity *v*_*l*_ where *l*-index takes the value 1 or 2 depending on the atom being considered. For counter-propagating configuration of the probe and coupling lasers with wave vector *k*_*P*_ and *k*_*C*_ respectively, the detunings are modified as $${{\rm{\Delta }}}_{P}={{\rm{\Delta }}}_{P}-{k}_{P}{v}_{l}$$ and $${{\rm{\Delta }}}_{C}={{\rm{\Delta }}}_{C}+{k}_{C}{v}_{l}$$ with $${\rm{\Delta }}k={k}_{C}-{k}_{P}$$. Thus the laser detunings from the two-atom transitions are modified as $${{\rm{\Delta }}}_{1}={{\rm{\Delta }}}_{C}+{k}_{C}{v}_{1}-{{\rm{\Omega }}}_{P}^{2}\mathrm{/4(}{{\rm{\Delta }}}_{P}-{k}_{p}{v}_{1})$$ and $${{\rm{\Delta }}}_{2}={{\rm{\Delta }}}_{P}+{{\rm{\Delta }}}_{C}+({\rm{\Delta }}k){v}_{2}+{{\rm{\Omega }}}_{P}^{2}\mathrm{/4(}{{\rm{\Delta }}}_{P}-{k}_{p}{v}_{2})$$. Doppler averaged Rydberg population can be determined by calculating the integral $${\rho }_{rr}=\frac{1}{\pi {v}_{p}^{2}}\int \int {\rho }_{rr}({v}_{1},{v}_{2}){e}^{-{v}_{1}^{2}/{v}_{p}^{2}}{e}^{-{v}_{2}^{2}/{v}_{p}^{2}}d{v}_{1}d{v}_{2}$$, with *v*_*p*_ being the most probable velocity. We have used a Monte-Carlo technique to evaluate the integral. The main peak at the two-photon resonance has to be calculated using the two-atom model by driving the atoms from the state |*g*_2_ *g*_2_〉 to the Rydberg state.

### Interacting dressed state model

Referring to Fig. 3, consider that the atom in the dressed state |*g*_1_〉 is placed at the center of the interaction sphere and the other atom in the dressed state |*g*_2_〉 is present in a concentric spherical shell with radius *r* and thickness *dr*. Resonance condition to the |*rr*〉 state constrain the velocity of the second atom to depend on the velocity of first atom as well as on their inter-particle separation *r*. If the resonant atoms within the line width of Rabi coupling are assumed to contribute significantly to the anti-blockade then the above constraint can be used to reduce the complexity of the model. Taking the vapor density as *η*, the number of atoms in the dressed state |*g*_2_〉 present inside the spherical shell with radius *r* is $\eta 4\pi {r}^{2}dr$. Suppose, only the velocity class of atoms at *v*_2_ within a small velocity width ${\rm{\Delta }}{v}_{2}={{\rm{\Omega }}}_{2}/{\rm{\Delta }}k$ inside the same spherical shell satisfy the resonant condition to the |*rr*〉 state, then the effective number of atoms inside the interaction sphere contributing to the anti-blockade can be evaluated as ${N}_{b}({v}_{1})=\frac{4\pi \eta }{\sqrt{\pi }{v}_{p}}\frac{{{\rm{\Omega }}}_{2}}{{\rm{\Delta }}k}{\int }_{0}^{{r}_{b}}{r}^{2}{e}^{-{v}_{2}^{2}/{v}_{p}^{2}}dr$ where *v*_2_ depends on *v*_1_ and *r*. In the case of probe laser detuning larger than the Doppler width, ${{\rm{\Delta }}}_{P}\gg {k}_{P}{v}_{2}$, the light shift of the |*g*_2_〉 state can be expanded to be $\mathrm{(1}+{k}_{P}{v}_{2}/{{\rm{\Delta }}}_{P}){{\rm{\Omega }}}_{P}^{2}\mathrm{/4}{{\rm{\Delta }}}_{P}$, neglecting the higher order terms. Then, the velocity of the second atom is found to be ${v}_{2}=(2{C}_{6}/{r}^{6}-{{{\rm{\Delta }}}_{1}}^{^{\prime} }({v}_{1}))/{\rm{\Delta }}k^{\prime}$, where ${{{\rm{\Delta }}}_{1}}^{^{\prime} }({v}_{1})={{\rm{\Delta }}}_{P}+2{{\rm{\Delta }}}_{C}+{k}_{C}{v}_{1}-{{\rm{\Omega }}}_{P}^{2}{k}_{P}{v}_{1}\mathrm{/4}{{\rm{\Delta }}}_{P}^{2}$ and ${\rm{\Delta }}k^{\prime} ={\rm{\Delta }}k+{{\rm{\Omega }}}_{P}^{2}{k}_{P}\mathrm{/4}{{\rm{\Delta }}}_{P}^{2}$. The above integral can be solved analytically to find the total number of atoms contributing to the anti-blockade as ${N}_{b}({v}_{1})=\frac{\pi \eta {{\rm{\Omega }}}_{2}\sqrt{8{C}_{6}}{\rm{\Delta }}k^{\prime} }{{\rm{\Delta }}k{({{{\rm{\Delta }}}_{1}}^{^{\prime} }({v}_{1}))}^{\frac{3}{2}}}$. Rydberg population can be related to the dispersion which is a measurable quantity using optical heterodyne detection technique as $\Re ({\chi }_{3L}({v}_{1}))=\frac{\eta |{\mu }_{eg}{|}^{2}{N}_{b}({v}_{1})}{{\varepsilon }_{0}\hslash ({{\rm{\Delta }}}_{P}-{k}_{p}{v}_{1})}{\rho }_{rr}({v}_{1})$^42^. Averaging over the velocity distribution of the first atom, the dispersion of the probe due to anti-blockade can be evaluated as

1 $$\Re ({\chi }_{3L})=\frac{{{\rm{\Omega }}}_{2}\sqrt{8\pi {C}_{6}}{\rm{\Delta }}k^{\prime} |{\mu }_{eg}{|}^{2}}{{\varepsilon }_{0}\hslash {v}_{p}{\rm{\Delta }}k}{\eta }^{2}{\int }_{-\infty }^{\infty }\frac{{\rho }_{rr}({v}_{1}){e}^{(-{v}_{1}^{2}/{v}_{p}^{2})}}{({{\rm{\Delta }}}_{P}-{k}_{P}{v}_{1})({{{\rm{\Delta }}}_{1}}^{^{\prime} }({v}_{1}{))}^{\frac{3}{2}}}d{v}_{1}$$
